# Auto-Brewery Syndrome: A Clinical Dilemma

**DOI:** 10.7759/cureus.10983

**Published:** 2020-10-16

**Authors:** Asim Tameez Ud Din, Faran Alam, Ahsan Tameez-ud-din, Farooq Mohyud Din Chaudhary

**Affiliations:** 1 Internal Medicine, Shifa International Hospital, Islamabad, PAK; 2 Internal Medicine, Rawalpindi Medical University, Rawalpindi, PAK; 3 Gastroenterology, Buch International Hospital, Multan, PAK; 4 Gastroenterology, Mohyud Din Clinic, Multan, PAK; 5 Gastroenterology, Nishtar Hospital, Multan, PAK

**Keywords:** auto brewery syndrome, ethanol, gut fermentation

## Abstract

Auto-brewery syndrome (ABS), also known as gut fermentation syndrome, is a very rare disorder. It is characterized by the endogenous production of alcohol. It typically presents with the signs of alcohol intoxication, such as staggering gait, slurred speech, gastrointestinal distress, and state of confusion. Due to the nonspecific symptoms, it is necessary to rule out other etiologies before reaching a diagnosis of ABS. The confirmatory test for this syndrome is the raised levels of blood or breath ethanol after a glucose challenge test. The management includes the use of antifungal drugs and avoidance of a carbohydrate-rich diet. In this review, we summarize the etiology, clinical presentation, diagnostic tests, management, and medicolegal aspects of ABS.

## Introduction and background

Alcohol is one of the oldest known beverages. It has an important role in the social life of a large percentage of the world’s population, although drinking habits vary widely across the world [1]. Owing to the wide-ranging social and medicolegal implications of alcohol consumption, the idea of its endogenous production is an area of fascination for investigators and the general public alike. Auto-brewery syndrome (ABS), also known as gut fermentation syndrome, describes the condition in which the concentration of ethanol increases to a noticeable level in the setting of little or no alcohol consumption [2]. It is a rare condition that is more prevalent in patients with underlying gut problems [2,3]. The suggested mechanism of this unique phenomenon revolves around the fermentation of carbohydrates in the human body by microorganisms [3].

The pharmacokinetics of alcohol is influenced by a variety of factors [4]. Ethanol undergoes first-pass metabolism before entering the bloodstream; therefore, the quantity of ethanol in the body should exceed 6 to 8 g per hour before it starts accumulating in the body fluids [5,6]. The endogenous production of ethanol in such a large amount is not supported by sufficient data, but the novelty of this unique phenomenon has given rise to many misconceptions and unreliable information that may be manipulated for medicolegal purposes [6].

This review article aims to improve the understanding of this rare condition by analyzing the findings of various case reports and studies. The main objective of this article is to give a comprehensive view of the different presentations of the ABS and its management.

## Review

Epidemiology and patient characteristics

ABS is being recognized in all age groups and in males as well as females. Al-Awadhi et al. conducted a study in the United Arab Emirates (UAE) to demonstrate the endogenous levels of blood ethanol in different subsets of the population. No significant difference was attributed to age, gender, and nationalities of the participants [7]. Similar results were replicated by Ragab et al. in the Saudi Arabia population [8]. Nair et al. in a study involving non-alcoholic steatohepatitis (NASH) patients found raised levels of breath alcohol concentration in obese and females, but the latter was not significant [9]. Another case-control study conducted by Cordell et al. included 52 diagnosed cases of ABS and revealed significant differences in the health status and daily life activities between the two groups. The striking contrast was in terms of gastrointestinal motility, intake of liquids, and sensitivities of food. Overall, the patients included in the ABS group were found to be less healthy and having more frequent bowel movements, higher sensitivity to foods, consumption of more water, and avoidance of starch-containing products [3].

Pathophysiology

ABS stems from the widespread proliferation of gut microorganisms, which, in turn, leads to endogenous production of ethanol. This phenomenon is preceded mostly by the intake of carbohydrate-rich meals or antibiotic use, which can disturb the gut ecosystem [2,10]. It is also frequently associated with underlying pathology. Hafez et al. demonstrated significantly higher concentrations of ethanol in diabetic (4.85 ± 3.96 mg/dL) and liver cirrhosis patients (3.45 ± 2.65 mg/dL) than the control group (0.3 ± 0.41 mg/dL) [11]. This syndrome is also found to be in patients suffering from other disorders such as Crohn’s disease, short bowel syndrome, and chronic intestinal pseudo-obstruction [12-15]. Though ethanol production and metabolism rates can differ in different subsets of the population owing to variations in dietary intake and genetic polymorphism of aldehyde dehydrogenase enzyme, its association with ABS warrants further studies [2,6,16].

Causative organisms

The causative organisms implicated in ABS include fungi and bacteria, with the most common yeasts being *Saccharomyces* and *Candida* species. A list of cases reported in the literature along with the involved microorganism is shown in Table 1. This list includes different species of *Saccharomyces* and *Candida* such s S. cerevisiae, *C. albicans*, *C. parapsilosis*, *C. glabrata*, *C. kefyr*, and *C. krusei *[12-15,17-23]. The involvement of these yeasts in the production of ethanol was validated by Bivin and Heinen. The four yeasts (*C. Albicans*, *C. tropicalis*, *S. cerevisiae*, *Torulopsis glabrata*) were mixed with different sugars, and the ethanol generated was measured by gas chromatography. All of them produced ethanol in all the test solutions highlighting the underlying etiology of ABS syndrome [24]. Some cases of bacterial involvement are also available in the literature [18]. Baraona et al. conducted the experiments on rats and demonstrated the ethanol formation by bacteria residing in the intestine [25].

**Table 1:** Reported Cases of Auto-Brewery Syndrome in the Literature IV, intravenous

Author	Year	Causative agent	Symptoms	Treatment	Resolution
Malik et al. [17]	2019	Saccharomyces cerevisiae, Candida albicans, Candida parapsilosis	Memory loss, mental changes, episodes of depression	IV micafungin	Yes
Saverimuttu et al. [18]	2019	Saccharomyces cerevisiae, Intermedia, Klebsiella pneumoniae, Enterococcus faecalis	Seizures, slurred speech, poor coordination	IV micafungin	Yes
Akhavan et al. [21]	2019		Slurred speech, fatigue, stumbling, dizziness, nausea	Oral fluconazole	Yes
Guo et al. [20]	2018	Candida parapsilosis	Unexplained gastrointestinal discomfort, intoxication	Initial fluconazole then switch to voriconazole plus nystatin	Yes
Welch et al. [12]	2016	Candida glabrata	Dizziness, slurred speech	changing diet to a low-carbohydrate diet, avoiding antibiotics	Yes
Cordell et al. [23]	2015	Candida albicans, Candida krusei	Episodes of drunkenness	Oral nystatin	Improved But not Completely Resolved
Cordell et al. [23]	2015	Saccharomyces cerevisiae	Loss of balance, slurred speech, passing out, GIT symptoms	Diet modification	Yes
Cordell et al. [23]	2015		Gastrointestinal Distress	Oral fluconazole	No
Cordell et al. [22]	2013	Saccharomyces cerevisiae	Excessively intoxicated after minimum or no drinking	Oral fluconazole	Yes
Jansson-Nettelbladt et al. [14]	2006	Candida kefyr, Saccharomyces cerevisiae	Walking erratically, bizarre behavior	Oral fluconazole	Yes
Spinucci et al. [15]	2006	Candida albicans, Saccharomyces cerevisiae	Abdominal pain, bloating, mental confusion	Oral fluconazole	Yes
Dahshan and Donovan [13]	2001	Candida glabrata, Saccharomyces cerevisiae.	Bizarre behavior, Somnolence, disorientation	Oral fluconazole	Yes
Kaji et al. [19]	1984	Candida albicans	Faintness, nausea, vomiting, comatose	Oral trichomycin B	Yes
Kaji et al. [19]	1984	Candida albicans	Difficulty in articulation, blurred vision, staggering gait	Oral trichomycin B	Yes

Clinical presentation and differentials

Patients with ABS mainly present with the signs of alcohol intoxication. These include neurological, gastrointestinal, respiratory, and psychological abnormalities. The neurologic symptoms mentioned in the literature include seizures, slurring of speech, incoordination leading to falls, blurring of vision, faintness, and memory loss [17-19]. The gastrointestinal abnormalities include nausea, vomiting, diarrhea, and generalized abdominal discomfort [20,23]. The psychological disorders including depression, bizarre behavior, somnolence, disorientation, fatigue, and a state of mental confusion are commonly associated with it. Respiratory symptoms such as runny nose and cough can also be seen in some patients [13,26]. Due to a wide range of nonspecific signs and symptoms, it is really important to rule out other etiologies such s head trauma, closet drinking, lactic acidosis, and other psychiatric disorders [2,27].

Diagnosis

The diagnosis of ABS can be a challenging aspect. The first step is a thorough history and examination. The complaints of intoxication signs without the intake of alcohol products can provide a diagnostic clue. The next step is laboratory investigations including baseline tests, metabolic profile, blood alcohol levels (BAL), and fecal testing for yeast growth. It is imperative to rule out other common etiologies with similar presentations including neurologic and psychiatric disorders. The confirmatory test for ABS syndrome is a glucose challenge test. There are two forms discussed in the literature: a standardized test that involves giving a specific amount of glucose and a nonspecific provision of carbohydrate meals [17]. Hunnisett et al. first described a method to detect endogenous alcohol production using a glucose challenge test. The first group was subjected to an oral glucose tolerance test with 50 g glucose after fasting overnight. The second group suspected of gut fermentation was given an oral glucose challenge test using 5 g glucose. Care was taken that the participants have not taken any alcohol for the last 24 hours and nothing by mouth for at least three hours. A rise in BAL of at least 0.5 mg/dL was considered positive. The majority of patients in both groups showed increased levels of blood ethyl alcohol (69% and 61%, respectively) following one hour, indicating fermentation in the gut [28]. Eaton summarized another diagnostic study in which control and test participants were given 1 g of glucose as a hard gelatin capsule followed by 4 g of glucose. The control group did not show an increase in BAL while the other group demonstrated a rise of 1-7 mg/dL in BAL [26]. In another standardized test, as discussed by Malik et al., 200 g of glucose is given at specific time intervals with regular monitoring of BAL. If BAL is raised within the first few hours, the test can be terminated. If not, we have to wait till 24 hours before ruling out ABS syndrome. The idea behind this time limit is based on the fact that it can take 24 hours for some fungi to ferment the carbohydrates. To analyze the underlying causative agent, gastrointestinal endoscopy is performed, and secretions from the stomach, small intestine, and cecum are used for culture and sensitivity [17]. Few cases discussed in the literature used nonspecific glucose challenge tests to diagnose this condition. This involves providing nonspecific carbohydrate meals to the suspected patient and analyzing blood and breath alcohol level [13,22].

Management

The management of ABS involves medical treatment as well as lifestyle modifications. If the patient presents with a severe state of alcohol intoxication, it should be managed as an emergency including airway, breathing, and fluids administration [2,29]. The medications that can be used for the treatment of ABS depend on the microorganism involved. As yeasts are commonly implicated in this syndrome, antifungals are frequently used. In our literature search, the common antifungals used for its treatment include fluconazole, nystatin, micafungin, trichomycin B, and voriconazole [13-15,17-21]. Antibiotics are used only if the involved agent is bacteria. Another important aspect of ABS management is diet modification. As carbohydrates are involved in the pathogenesis of this syndrome, patients are advised to limit the intake of carbohydrates and increase protein consumption [2]. Some reported cases in the literature were improved only by following the dietary guidelines and there was no need for drug therapy [12]. There is also a use of probiotics such as Lactobacillus in some cases, but its efficiency in this syndrome is yet to be studied [18].

Complications

Although the psychological impact of endogenous ethanol production has been recognized, there are a few other complications mentioned in the literature [2]. Zhu et al. advocated that there are certain gut bacteria involved in the production of endogenous alcohol, which, in turn, can be attributed to the development of NASH [30]. Eaton et al. highlighted the effect of gut fermentation on vitamins and minerals. Vitamin B6 was found to be most affected by this syndrome. Minerals such as zinc and magnesium were also found to be affected by this phenomenon [31]. The role of ABS in sudden infant death syndrome (SIDS) has also been found in the literature, but there is little evidence to support this argument [32].

Relation with drugs

An interesting correlation between the cimetidine and ethanol production in the stomach was demonstrated by Bode et al. The participants who received either cimetidine or antacids manifested higher ethanol levels in the gastric juice as compared to the other group. Though it needs further evidence to evaluate the exact etiology, a change in pH caused by these drugs led to the proliferation of microorganisms which in turn was responsible for ethanol production [33].

Medicolegal aspect

The medicolegal aspect of ABS syndrome is an interesting but controversial topic in the field of forensic medicine. The values of BAL and breath alcohol levels are sometimes challenged in the court, and the defense rests on the idea of endogenous production of alcohol by ABS syndrome. Multiple studies have been conducted to demonstrate the normal levels of alcohol that can be produced in the body. The levels were detected in different bodily fluids such as blood, urine, and plasma, as well as breath, using sensitive tests including gas chromatography and enzymatic oxidation. These studies showed that although there is a small amount of alcohol produced in the body, the probability of endogenous production of relatively high levels (>80 mg/dL) is quite rare with ABS syndrome [6]. Simic et al. conducted a study to analyze the relation between diabetes mellitus and the endogenous production of ethanol. The mean BAL were significantly greater in the diabetic group as compared to the control group (2.65 mg/dL vs 0.40 mg/dL) using the headspace gas chromatography method (HS-GC). BAL using a less specific test, the Widmark method, showed a higher mean value in diabetic patients (27.28 mg/dL), but this is still quite lower than the levels for illegal intoxication. Urine alcohol levels were also found to be significantly higher in the diabetic group (6.13 mg/dL vs 3.27 mg/dL) measured by the HS-GC method. using Widmark’s method, relatively higher values were seen between the diabetic and control group (54.27 mg/dL vs 8.30 mg/dL) [34]. In contrast to this, a study was conducted by Alexander et al. in 1988 to compare the urine ethanol levels of 10 diabetic patients with the urine of a healthy volunteer. Initially, the levels of ethanol were minimal as detected by gas-liquid chromatography, but a considerable large amount of ethanol was found in diabetic patients over a period of three weeks (about 700 mg/dL on the third day in one patient) [35]. In different cases reported in the literature, a high level of BAL was found, leading to intoxication signs. Considering the variability in the literature, more studies are required to reach a conclusion on this aspect.

## Conclusions

ABS is a rare disorder with nonspecific signs and symptoms. Avoidance of a carbohydrate-rich diet and other simple remedies are the mainstays of its management. A wider understanding of this disorder is needed in order to avoid unnecessary legal misunderstandings. This can be achieved simply by proper history-taking and a thorough examination of the patient, which can help avoid diagnostic uncertainties and make timely healthcare decisions.
